# Depressive disorders in Brazil: results from the Global Burden of Disease Study 2017

**DOI:** 10.1186/s12963-020-00204-5

**Published:** 2020-09-30

**Authors:** Cecília Silva Costa Bonadiman, Deborah Carvalho Malta, Valéria Maria de Azeredo Passos, Mohsen Naghavi, Ana Paula Souto Melo

**Affiliations:** 1 Faculdade de Medicina, Programa de Pós- Graduação em Saúde Pública, Universidade Federal de Minas Gerais, Belo Horizonte, MG Brazil; 2 Departamento de Enfermagem Materno Infantil e Saúde Pública, Escola de Enfermagem, Universidade Federal de Minas Gerais, Belo Horizonte, MG Brazil; 3 Faculdade de Ciências Médicas de Minas Gerais, Belo Horizonte, MG Brazil; 4 Institute for Health Metrics and Evaluation, Seattle, WA USA; 5 Faculdade de Medicina, Universidade Federal de São João Del Rei, Divinópolis, MG 35501-296 Brazil

**Keywords:** Mental health, Mental disorders, Depressive disorders, Disability-adjusted life years, Descriptive epidemiology

## Abstract

**Background:**

Depression is one of the major causes of disability worldwide. The objective of this study was to analyze the results of the Global Burden of Disease Study 2017 (GBD-2017) for depressive disorders in Brazil and its Federated Units (FUs) in 1990 and 2017.

**Methods:**

We used GBD-2017 study methodology to evaluate the prevalence estimates, the disability-adjusted life-year (DALY), and the years lived with disability (YLDs) for depressive disorders, which include major depressive disorder and dysthymia. The YLD estimates and the position of these disorders in the DALY and YLD rankings were compared to those of seven other countries. The observed versus expected YLD, based on the sociodemographic index (SDI), were compared.

**Results:**

In GBD-2017, the prevalence of depressive disorders in Brazil was 3.30% (95% uncertainty interval [UI]: 3.08 to 3.57), ranging from 3.79% (3.53 to 4.09) in Santa Catarina to 2.78% in Pará (2.56 to 3.03), with significant differences between the Federated Units. From 1990 to 2017, there was an increase in number of YLD (55.19%, 49.57 to 60.73), but a decrease in the age-standardized rates (− 9.01%, − 11.66 to − 6.31). The highest proportion of YLD was observed in the age range of 15–64 years and among females. These disorders rank 4th and 13th as leading causes of YLD and DALY, respectively, in Brazil. In the other countries evaluated, the ranking of these disorders in the YLD classification was close to Brazil’s, while in the DALY classification, there was higher variability. All countries had YLD rates similar to the overall rate. The observed/expected YLD ratio ranged from 0.81 in Pará to 1.16 in Santa Catarina. Morbidity of depressive disorders was not associated with SDI.

**Conclusions:**

Depressive disorders have been responsible for a high disability burden since 1990, especially in adult women living in the Southern region of the country. The number of people affected by these disorders in the country tends to increase, requiring more investment in mental health aimed at advancements and quality of services. The epidemiological studies of these disorders throughout the national territory can contribute to this planning and to making the Brazilian health system more equitable.

## Background

Depressive disorders are responsible for significant personal and family suffering, functional impairment, high costs for health, and social security systems [1, 2] and are associated with premature mortality from suicide and other diseases [1]. Besides being one of the most prevalent mental disorders in the world, depression has been reported as one of the major contributors to burden of disease worldwide since the first publication of the Global Burden of Disease Study (GBD), in the 1990s [3]. This confirms the need to prioritize depressive disorders and mental disorders in the global public health agendas [4].

The latest GBD study publication, GBD-2017, described 359 causes of diseases and injuries in 195 countries from 1990 to 2017. GBD provides a standardized methodology of data analysis, which allows data comparability among and within countries, to the impact of fatal and nonfatal conditions, as depressive disorders. GBD Data is recalculated each new publication, allowing trends to be investigated on a regular basis [5].

In GBD studies, burden of disease is measured in terms of disability-adjusted life-years (DALY), a composite indicator that results from the sum of years lived with disability (YLD) and years of life lost due to premature mortality (YLL) [3]. In GBD-2017, depressive disorders contributed with 1.72% (95% UI: 1.3–2.19) of all global DALY and were considered the 15th leading cause of DALY [5] and the 3rd leading cause of disability (YLD), and accounted for 5.05% (95% UI: 4.15–6.11) of the total YLD in the world [6]. An in-depth review of burden due to depressive disorders using latest GBD-2017 results has yet to be performed in Brazil.

GBD morbidity estimates are based on a systematic review of the literature to obtain all available epidemiological data on each health outcome [5, 6]. In Brazil, there are few representative population studies with data on prevalence of depressive disorders. The most recent of these studies, the National Health Survey (*Pesquisa Nacional de Saúde*—PNS-2013), with data from all Federated Units (FUs) [7], showed a point prevalence of depression of 4.1% (95% confidence interval [CI] 3.8–4.4) and significant differences in prevalence among the regions of the country [8]. In a systematic review and meta-analysis not including the PNS 2013, Silva et al. [9] found a prevalence of depressive symptoms in the Brazilian population of 14% (95% CI 13–16), a prevalence in the last year of major depressive disorder (MDD) of 8% (95% CI 7–10), and a lifelong prevalence of the MDD of 17% (95% CI 14–19). However, the samples from the 27 studies included in this review were selected mainly in the Southeastern and Southern regions, which are the richest in the country and probably did not reflect the reality of the less developed regions of Brazil. In addition, methodological variability between studies using different types of sampling and measuring instruments hinder data comparability.

Therefore, there is a need to update the burden of disease estimate for depressive disorders in Brazil and its FUs, considering the GBD 2017 results could improve the estimates in a continental-size country with great socioeconomic and cultural diversity. The objective of this study was to use GBD 2017 estimates to analyze the burden of depressive disorders in Brazil and FUs, according to sex and age, in 1990 and 2017, in addition to comparing Brazil's estimates with those of other seven countries. Furthermore, we present the relationship between the socio-demographic index (SDI) and depressive disorder YLDs in 2017.

## Methods

A descriptive study was carried out with estimates based on secondary data on the burden of depressive disorders for Brazil in the study GBD-2017, coordinated by the Institute for Health Metrics and Evaluation (IHME), University of Washington, United States of America (USA) [5]. All metrics were estimated separately for the 27 FUs in Brazil. The GBD follows the Guidelines for Accurate and Transparent Health Estimates Reporting (GATHER Statement).

In GBD-2017, depressive disorders were divided into two subtypes, defined according to the diagnostic criteria of the Diagnostic and Statistical Manual of Mental Disorders-revised version of the fourth edition (DSM-IV-TR) [10] or their equivalent diagnoses in the International Statistical Classification of Diseases and Related Health Problems (ICD-10) [11]. The disorders comprise MDD (DSM-IV-TR: 296.21-24, 296.31-3; ICD-10: F32.0-9, F33.0-9) and dysthymia (DSM-IV-TR: 300.4; ICD-10: F34.1). In the present study, estimates of MDD and dysthymia will be presented as a single category called depressive disorders.

According to DSM-IV-TR, MDD is an episodic mood disorder and involves the presence of at least one major depressive episode (MDE). The diagnosis requires the presence of five or more of the following symptoms for at least 2 weeks, including necessarily, depressed mood or anhedonia associated with change in eating, appetite, or weight, excessive sleeping or insomnia, agitated or slow motor activity, low energy or fatigue, feeling worthless or inappropriately guilty, trouble concentrating, and repeated thoughts about death [10].

Dysthymia is described in DSM-IV-TR as a type of chronic depression, with symptoms less severe than MDD, but long-lasting. The diagnosis requires depressed mood to be present for at least 2 years (or at least 1 year in children and adolescents), plus two of the following symptoms in the same period: poor appetite or overeating, insomnia or hypersomnia, low energy or fatigue, low self-esteem, poor concentration or indecisiveness, and feelings of hopelessness [10].

For the depressive disorders, DALY was based exclusively on YLD, because these disorders are not considered by ICD-10 [11] as a direct cause of death. Thus, in this article, we will use the YLD as the main indicator, since its values are the same for DALY.

Age-standardized rates per 100,000 inhabitants produced by the direct standardization method, with the world population developed for GBD [5] as the standard, are presented in this study. For all estimates, 95% uncertainty intervals (95% UI) were considered.

The calculation of YLDs was performed by multiplying two components: the prevalence of depressive disorders and the disability weight, reflecting the loss of health associated with depressive disorders on a scale of 0 (perfect health) to 1 (equivalent to death). These disability weights quantify the severity of loss of health associated with each sequela, or consequence of disease/injury [6].

The same disability weights estimated in GBD-2013 were used in GBD-2017. The disability weights were obtained through face-to-face surveys in nine countries and complemented by a web survey involving 60,890 respondents from 167 countries. Participants were lay individuals who had to choose between two descriptions of health states they considered to be more disabling [6].

Disability weights were estimated for each level of severity of MDD: mild (0.145; 95% CI 0.099–0.209), moderate (0.396, 95% CI 0.267–0.531), and severe (0.658; 95% CI 0.477–0.807). For dysthymia, it was assigned the same disability weight as that for mild MDD (0.145). The proportion of people at each MDD severity level was also estimated: asymptomatic (13%; 95% CI 10–17%), mild (59%, 95% CI 49–69%), moderate (17%; 95% CI 13–22%), and severe (10%, 95% CI 3–20%). In the case of dysthymia, 29% (95% CI 23–36%) were considered asymptomatic and 71% were symptomatic (95% CI 64–77%). A more comprehensive explanation of the methods for quantifying disability weights and to determine the proportion of cases at each level of severity is available elsewhere [6].

Prevalence was obtained using data from Brazilian population-based studies, totaling 18 sources of information, described in Table 1, which can be accessed at http://ghdx.healthdata.org/gbd-2017/data-input-sources. Two out of the total data sources used [7, 18] consist of public database access. The other ones are publications of scientific papers. These studies were selected through a systematic literature review, on a peer-review basis, following the guidelines recommended by the Preferred Reporting Items for Systematic Reviews and Meta-Analyzes (PRISMA) [29], in the electronic databases PsycInfo, Embase, and PubMed.

**Table 1:** Characteristics of the studies used to estimate the burden of depressive disorders in Brazil, GBD-2017

Author(s)	Location of studies	Area	Age range (years)	Instruments	Sample size	% of women
Andrade et al. 2002 [12]	São Paulo (Catchment area study)	Urban	≥ 18	CIDI	1464	57.5
Andrade et al. 2012 [13]	São Paulo (Megacity study)	Urban	≥ 18	CIDI	5037	56.6
Kessler et al. 2010 [14]	São Paulo (Megacity study)	Urban	≥ 18	CIDI	5037	56.6
Anselmi et al. 2010 [15]	Pelotas -RGS (Brazilian Birth Cohort Study)	Urban	≥ 18	SDQ DAWBA	4452	50.3
Bahls, 2002 [16]	Curitiba—PR	Urban	10–17	CDI	463	58.1
Barcelos-Ferreira et al. 2013 [17]	São Paulo—SP	Urban	≥ 60	D-10 CAMDEX CAMCOG	1563	68.7
Brazilian Institute of Geography and Statistics (IBGE), 2013 [7]	Brazil (PNAD)	Mixed	≥ 18	PHQ-9	60202	52.9
Center for Scientific and Technological Information, Oswaldo Cruz Foundation and World Health Organization (WHO), 2005 [18]	Brazil (WHS)	Mixed	≥ 18	Version of CIDI	5000	51.54
Chiavegatto Filho et al. 2013 [19]	São Paulo (Megacity study)	Urban	≥ 18	CIDI	3542	55.9
Coelho et al. 2013 [20]	Brazil (Brazilian alcohol survey)	Mixed	≥ 14	CES-D	3007	52.9
Costa et al. 2007 [21]	Bambuí—MG (BHAS)	Urban	≥ 75	GHQ-12 GDS-30 MMSE	392	62.5
Da Silva et al. 2013 [22]	São Paulo (São Paulo Ageing and Health Study)	Urban	≥ 65	GMS NPI	2072	39.4
Fleitlich-Bilyk et al. 2004 [23]	Taubaté—SP	Mixed	7-14	DAWBA	1251	47
Lopez et al. 2011 [24]	Pelotas—RGS	Urban	18-24	MINI 5.0	1560	56.4
Petresco et al. 2014 [25]	Pelotas -RGS (2004 Pelotas Birth Cohort)	Urban	6	DAWBA	3585	48.7
Ribeiro et al. 2013 [26]	Rio de Janeiro and São Paulo	Urban	15-75	CIDI	3744	56.7
Salum et al. 2015 [27]	São Paulo and Porto Alegre (High risk cohort study)	Urban	6-12	DAWBA	2512	46.9
Vorcaro et al. 2001 [28]	Bambuí—MG (BHS)	Urban	≥ 18	CIDI	1041	56.5

*BHAS* Bambuí Health Ageing Study, *BHS* Bambuí Health Survey, *CAMDEX* Cambridge Mental Disorders of the Elderly Examination, *CAMGOG* Brief neuropsychological testing (cognitive section of the CAMDEX), *CDI* Children’s Depression Inventory, *CES*-*D* Center for Epidemiologic Studies Depression Scale, *D*-*10* Brief instrument for screening of depressive disorders in elderly people, *DAWBA* Development and Well-Being Assessment, *GDS*-*30* Geriatric Depression Scale, *GHQ*-*12* General Health Questionnaire, *GMS* Geriatric Mental Status, *MINI* Mini-International Neuropsychiatric Interview, *MG* Minas Gerais, *MMSE* Mini-Mental State Examination, *NPI* Neuropsychiatric Inventory, *PNAD Pesquisa Nacional por Amostra de Domicílios* (National Household Sample Survey), *PHQ*-*9* Patient Health Questionnaire, *PR* Paraná, *RGS* Rio Grande do Sul, *RJ* Rio de Janeiro, *SDQ* Strengths and Difficulties Questionnaire, *SP* São Paulo, *WHS* World Health Survey

Studies in any given language were taken, considering the following inclusion criteria (1) studies published as from 1980; (2) case definition based on the clinical threshold established by the DSM or ICD, assessed by diagnostic instruments or symptom scales; (3) study with sufficient information about the method and characteristics of the sample to evaluate its quality; and (4) study samples should represent the general population (i.e., samples of inpatient or drug treatment, case studies, veterans, or samples of refugees were excluded). As for the prevalence measures, last year or point estimates were required. Although point prevalence is the most representative measure for GBD purposes, since it measures actual disability, the prevalence of the last/past year was accepted to maximize inclusion. Life-long estimates were excluded because they are more susceptible to memory bias. As for cases where the same data were reported in different papers, the most informative one was selected [6].

In addition to these data, an important advance has been incorporated, which consists in the attribution of a proportion of cases of suicide due to MDD. The data were modeled in DisMod-MR 2.1, a Bayesian meta-regression tool, which generates consistent estimates of incidence, prevalence, remission duration, and excess risk of death for both sexes, age groups, year, and location [6].

Throughout the modeling phase, a number of adjustments were made to improve the predictive power of the prevalence model. Three years old was considered the minimum age for the manifestation of depressive disorders, based on literature and experts feedback. In addition, covariates were used to minimize the methodological heterogeneity of the raw data set, adjusting sub-optimal estimates for optimal estimates. For example, symptom scales and last year prevalence data (sub-optimal estimates) were adjusted to the level of estimates derived from diagnostic instruments and based on point prevalence (optimal estimates). More details on the methodology have already been published [6].

The GBD also produces the SDI to measure the level of development of each country/subnational region. The SDI is the average of three indicators: total fertility rate, income per capita, and average education of the population over 15 years. SDI scores range from 0 (lower income, lower education, and higher fertility) to 1 (higher income, higher education, and lower fertility). Based on the SDI, it is possible to compare the data between locations with similar socioeconomic status [30].

In this study, the following indicators were described: age-standardized prevalence, absolute number, and YLD rate, by select countries besides Brazil and its FUs, in 1990 and 2017. Temporal change was evaluated by the difference of values between the time periods. Differences were considered statistically significant where the 95% UI did not include zero. YLD was described by age groups and sex.

The rank of DALY and YLD by depressive disorders in Brazil was compared to the global classification and that of seven countries: countries with similar socioeconomic situation and/or geographical proximity in Latin America (Mexico, Argentina, and Colombia), high SDI countries with public health system (Canada, Australia, and England), and the USA, a high SDI country with a private health system, with one of the highest prevalence of depression in the world. We also compared observed versus expected YLD in Brazil and FUs in 2017, based on by SDI-rates of depressive disorders. This ratio allows us to assess whether health outcomes were better or worse than would be expected based on SDI.

The Project “Global Burden of Disease Study—GBD Brazil” was approved by the Research Ethics Committee of the Universidade Federal de Minas Gerais (UFMG), under protocol number (CAAE Project—62803316.7.0000.5149).

## Results

The GBD-2017 included 18 studies to estimate the depressive disorders for Brazil. Of these, 15 [12–17, 19, 21–28] were carried out with samples from states in the Southern and Southeastern regions, and three [7, 18, 20] with national representativeness samples, including PNS-2013. Four studies were conducted with urban-rural samples [7, 18, 20, 23] and the remaining with urban samples. There was a wide variety in age groups: four studies evaluated exclusively children and adolescents [16, 23, 25, 27], three studies assessed only the elderly [17, 21, 22], and eight studies included people who were 18 years old and older [7, 12–15, 18, 19, 28]. Two studies evaluated adolescents and adults (≥ 14 years [20], and 15 to 75 years [26]) and another considered only young adults aged 18 to 24 years [24]. Two studies had fewer than 1000 participants [16, 21]. Depression was assessed by diagnostic instruments or symptom scales (Table 1).

In GBD-2017, the age-standardized prevalence of depressive disorders in both sexes in Brazil was 3.3% (95% UI: 3.08–3.57) with 7.2 million (7.7–6.7) cases; in that, 2.32% (95% UI: 2.13–2.53) and 1.04% (95% UI: 0.91–1.2) for MDD and dysthymia, respectively. In 2017, the highest prevalence was observed in the FUs of Santa Catarina (3.79%, 95% UI: 3.53–4.09), Rio Grande do Sul (3.67%, 95% UI: 3.43–3.97), and Roraima (3.67%, 95% UI: 3.41–3.97), while the lowest ones were observed in the FUs Pará (2.78%, 95% UI: 2.56–3.03), Bahia (2.96%, 95% UI: 2.74–3.23), and Amazonas (3.0%, 95% UI: 2.78–3.26). In 1990, there were 4.5 million (4.9–4.1) cases and the age-standardized prevalence in Brazil was 3.53% (95% UI: 3.28–3.84), with a decrease by 6.71% (95% UI: − 9.15 to − 4.09), between 1990 and 2017 (Table 2).

**Table 2:** Absolute number, rate, prevalence, and percentage of change of depressive disorders, 1990–2017, Brazil

Location	Number of YLD (UI95%)		% change 1990-2017	Age-standardized YLD rates (UI95%)		% change 1990–2017	Prevalence (UI95%)		% change 1990–2017
	1990	2017		1990	2017		1990	2017	
Global	28.251.850.21 (20.059.412.01 to 38.668.606.16)	43.099.909.40 (30.536.443.27 to 58.895.581.87)	52.56 (49.49 to 55.66)	565.78 (402.87 to 771.56)	540.45 (382.35 to 737.83)	− 4.48* (− 5.47 to − 3.45)	3.54 (3.3 to 3.84)	3.44 (3.21 to 3.73)	− 2.84* (− 3.7 to − 1.97)
Brazil	798.928.86 (562.340.59 to 1.095.631.47)	1.239.852.03 (878.911.25 to 1.689.498.88)	55.19 (49.57 to 60.73)	597.84 (423.19 to 810.05)	543.96 (386.79 to 740.75)	− 9.01* (− 11.66 to − 6.31)	3.53 (3.28 to 3.84)	3.3 (3.08 to 3.57)	− 6.71* (− 9.15 to − 4.09)
South region									
Santa Catarina	28.022.16 (19.445.13 to 38.032.11)	49.758.80 (34.942.68 to 67.691.45)	77.57 (66.31 to 88.92)	675.28 (469.66 to 911.84)	638.29 (447.04 to 868.23)	− 5.48 (− 10.64 to 0.19)	3.94 (3.63 to 4.31)	3.79 (3.53 to 4.09)	− 3.74 (− 8.25 to 1.2)
Rio Grande do Sul	60.465.82 (42.463.84 to 83.056.69)	78.645.96 (55.435.75 to 106.069.82)	30.07 (22.33 to 38.08)	679.74 (475.33 to 927.94)	611.57 (432.41 to 828.6)	− 10.03* (− 14.97 to − 4.65)	3.98 (3.67 to 4.33)	3.67 (3.43 to 3.97)	− 7.65* (− 12.18 to − 2.6)
Paraná	48.270.30 (33.749.78 to 65.874.09)	68.056.45 (47.764.26 to 92.965.54)	40.99 (32.64 to 49.95)	615.08 (433.18 to 830.87)	547.86 (385.85 to 750.12)	− 10.93* (− 15.6 to − 5.94)	3.64 (3.35 to 3.95)	3.33 (3.11 to 3.61)	− 8.28* (− 12.73 to − 3.65)
Southeast region									
São Paulo	193.105.95 (136.300.75 to 262.716.54)	276.749.85 (196.253.39 to 375.594.95)	43.32 (36.15 to 51.45)	637.71 (450.46 to 863.95)	556.89 (396.16 to 753.28)	− 12.67* (− 17.05 to − 8.10)	3.75 (3.48 to 4.05)	3.37 (3.13 to 3.64)	− 10.01* (− 14.02 to − 6.11)
Minas Gerais	86.836.31 (60.371.39 to 118.358.32)	130.221.78 (91.535.02 to 177.155.53)	49.96 (40.39 to 60.02)	597.97 (421.04 to 808.0)	550.21 (388.65 to 749.46)	− 7.99* (− 13.37 to − 2.57)	3.53 (3.26 to 3.86)	3.32 (3.08 to 3.58)	− 6.18* (− 10.8 to − 1.47)
Rio de Janeiro	75.198.85 (53.223.41 to 103.373.35)	98.441.09 (70.443.84 to 133.802.19)	30.91 (23.38 to 39.68)	583.17 (410.74 to 804.44)	503.58 (357.26 to 688.02)	− 13.65* (− 18.47 to − 8.49)	3.46 (3.18 to 3.79)	3.08 (2.84 to 3.35)	− 10.85* (− 14.94 to − 6.58)
Espírito Santo	12.872.09 (9.044.36 to 17.611.82)	22.668.85 (16.080.59 to 30.663.69)	76.11 (65.99 to 87.36)	550.2 (392.61 to 748.81)	535.33 (379.13 to 721.76)	− 2.7 (− 7.53 to 2.52)	3.31 (3.04 to 3.61)	3.26 (3.02 to 3.56)	− 1.55 (− 5.51 to 1.44)
Center West region									
Goiás	23.528.59 (16.383.92 to 32.068.5)	41.546.43 (29.463.49 to 56.335.61)	76.58 (65.8 to 87.77)	641.93 (452.03 to 865.67)	567.14 (402.34 to 770.48)	− 11.65* (− 16.61 to − 6.51)	3.76 (3.48 to 4.09)	3.42 (3.18 to 3.71)	− 8.94* (− 13.25 to − 4.49)
Mato Grosso do Sul	9.703.75 (6.769.72 to 13.269.6)	16.526.16 (11.646.83 to 22.384.9)	70.31 (59.41 to 83.14)	612.17 (427.27 to 830.14)	557.98 (390.89 to 756.38)	− 8.85* (− 14.16 to − 3.02)	3.61 (3.32 to 3.97)	3.38 (3.15 to 3.67)	− 6.36* (− 11.08 to − 1.28)
Distrito Federal	8.038.09 (5.587.76 to 1.1057.86)	16.507.89 (11.726.44 to 22.412.78)	105.37 (92.38 to 119.47)	562.55 (394.61 to 765.84)	523.68 (372.01 to 711.52)	− 6.91* (− 11.9 to − 1.91)	3.39 (3.11 to 3.72)	3.22 (2.98 to 3.49)	− 5.07* (− 9.5 to − 0.77)
Mato Grosso	9.091.56 (6.392.64 to 12.465.09)	19.129.98 (13.598.13 to 26.008.87)	110.41 (96.78 to 125.30)	536.92 (378.95 to 729.02)	516.99 (365.9 to 700.92)	− 3.71 (− 8.64 to 1.96)	3.23 (2.96 to 3.53)	3.17 (2.94 to 3.45)	− 1.92 (− 6.23 to 2.8)
Northeast region									
Pernambuco	39.079.57 (27.475.59 to 53.235.38)	61.005.65 (43.080.33 to 82.490.09)	56.11 (47.52 to 65.26)	617.55 (435.12 to 838.2)	590.65 (416.55 to 797.83)	− 4.36 (− 9.36 to 0.82)	3.63 (3.35 to 3.97)	3.52 (3.29 to 3.83)	− 2.82 (− 7.27 to 1.78)
Alagoas	12.158.09 (8.606.19 to 16.515.99)	20.661.48 (14.577.85 to 28.061.33)	69.94 (60.37 to 79.84)	574.49 (407.67 to 780.78)	579.94 (407.84 to 785.47)	0.95 (− 4.09 to 6.18)	3.41 (3.14 to 3.73)	3.48 (3.22 to 3.77)	1.96 (− 2.39 to 6.5)
Rio Grande do Norte	12.718.78 (8.908.37 to 17.331.3)	21.418.85 (15.081.41 to 29.134.88)	68.4 (58.34 to 79.08)	597.39 (420.14 to 811.71)	563.52 (396.16 to 767.47)	− 5.67 (− 10.77 to 0.29)	3.52 (3.25 to 3.85)	3.4 (3.16 to 3.68)	− 3.59 (− 7.98 to 1.44)
Ceará	32.034.30 (22.513.75 to 43.845.02)	56.766.75 (40.250.96 to 76.906.24)	77.21 (66.82 to 88.11)	577.31 (409.62 to 786.94)	563.73 (399.16 to 762.93)	− 2.35 (− 8.05 to 3.35)	3.42 (3.13 to 3.74)	3.39 (3.16 to 3.68)	− 0.75 (− 5.48 to 4.57)
Sergipe	7.426.69 (5.206.42 to 10.223.2)	13.138.65 (9.296.79 to 18.014.21)	76.91 (66.05 to 87.99)	588.36 (418.25 to 804.76)	538.49 (380.71 to 735.43)	− 8.48* (− 13.83 to − 2.96)	3.49 (3.21 to 3.82)	3.27 (3.04 to 3.55)	− 6.13* (− 10.73 to − 1.32)
Paraíba	15.590.88 (10.972.35 to 21.249.8)	23.650.99 (16.790.76 to 31.997.1)	51.7 (42.57 to 61.03)	550.8 (387.86 to 753.54)	533.97 (379.31 to 723.25)	− 3.06 (− 8.16 to 2.64)	3.63 (3.03 to 3.61)	3.52 (3 to 3.51)	− 1.68 (− 6.11 to 3.36)
Maranhão	21.912.07 (15.400.65 to 29.861.66)	39.418.39 (28.019.67 to 53.414.56)	79.89 (69.32 to 91.38)	551.8 (391.57 to 743.7)	522.13 (371.9 to 708.39)	− 5.38 (− 10.43 to 0.27)	3.29 (3.03 to 3.6)	3.18 (2.94 to 3.45)	− 3.28 (− 7.69 to 1.55)
Piauí	12.037.69 (8.427.93 to 16.367.59)	18.896.44 (13.323.91 to 25.721.69)	56.98 (46.87 to 68.41)	553.39 (392.26 to 752.54)	516.7 (364.24 to 701.92)	− 6.63* (− 12.05 to − 0.86)	3.32 (3.04 to 3.63)	3.16 (2.93 to 3.45)	− 4.67 (− 9.47 to 0.36)
Bahia	49.226.48 (34.521.76 to 67.953.22)	77.703.93 (54.936.49 to 105.557.79)	57.85 (46.20 to 68.72)	487.87 (343.84 to 664.84)	477.36 (336.67 to 648.52)	− 2.16 (− 8.6 to 4.65)	2.98 (2.73 to 3.29)	2.96 (2.74 to 3.23)	− 0.75 (− 5.99 to 4.36)
North region									
Roraima	1.092.80 (764.72 to 1.508.61)	3.381.03 (2.374.07 to 4.606.57)	209.39 (189.55 to 231.13)	641.03 (449.82 to 870.6)	618.68 (435.42 to 838.46)	− 3.49 (− 8.87 to 2.12)	3.72 (3.43 to 4.05)	3.67 (3.41 to 3.97)	− 1.16 (− 5.92 to 3.84)
Tocantins	4.201.43 (2.938.49 to 5.739.95)	9.090.10 (6.370.89 to 12.330.47)	116.36 (102.74 to 130.22)	564.51 (395.22 to 769.32)	560.08 (393.83 to 758.99)	− 0.78 (− 6.2 to 4.27)	3.37 (3.09 to 3.67)	3.38 (3.13 to 3.64)	0.32 (− 4.4 to 5.02)
Rondônia	5.429.99 (3.809.3 to 7.472.88)	9.709.04 (6.816.85 to 13.230.37)	78.8 (67.56 to 89.26)	587.45 (411.8 to 803.08)	533.31 (374.76 to 725.23)	− 9.22* (− 13.94 to − 4.35)	3.44 (3.16 to 3.75)	3.23 (2.99 to 3.5)	− 6.08* (− 10.42 to − 1.61)
Amapá	1.107.24 (769.75 to 1.525.25)	4.113.08 (2.911.07 to 5.576.96)	271.47 (249.12 to 294.73)	522.01 (368.42 to 717.81)	516.78 (366.4 to 699.12)	− 1 (− 6.33 to 4.63)	3.15 (2.88 to 3.44)	3.15 (2.92 to 3.42)	0.06 (− 4.39 to 5.02)
Acre	1.686.33 (1.176.84 to 2.301.14)	4.389.38 (3.094.54 to 5.942.43)	160.29 (144.14 to 176.65)	515.89 (367.46 to 701.33)	506.06 (360.92 to 686.53)	− 1.9 (− 7.26 to 3.06)	3.09 (2.84 to 3.38)	3.08 (2.85 to 3.34)	− 0.07 (− 4.73 to 4.64)
Amazonas	8.609.20 (96.038.25 to 11.830.41)	19.146.02 (13.569.46 to 26.095.45)	122.39 (106.9 to 137.35)	523.51 (372.35 to 712.24)	484.87 (344.08 to 659.49)	− 7.38* (− 12.77 to − 1.87)	3.15 (2.9 to 3.46)	3.00 (2.78 to 3.26)	− 4.95* (− 9.54 to − 0.11)
Pará	19.483.86 (13.698.42 to 26.910.79)	39.109.06 (27.682.13 to 53.227.52)	100.73 (87.5 to 114.95)	491.55 (350.15 to 670.37)	442.9 (315.81 to 600.09)	− 9.9* (− 15.28 to − 4.2)	2.99 (2.72 to 3.3)	2.78 (2.56 to 3.03)	− 6.95* (− 11.7 to − 1.98)

*YLD* years lived with disability, *UI* uncertainty intervals

Data in parentheses are 95% uncertainty intervals

Age-standardized YLD rates per 100 000 cases, both sexes, prevalent cases (percent), age-standardized, both sexes

* Rates with statistically significant changes by uncertainty intervals

Depressive disorders accounted for 1239 million (95% UI: 878.911–1.689.498) YLDs in Brazil in 2017, with a rate of 543.96 per 100,000 (95% UI: 386.79–740.75), accounting for 5% (95% UI: 4.04–6.09) of all YLDs in the country. Between 1990 and 2017, the age-standardized YLD rate decreased by 9.01% (95% UI: − 11.66 to − 6.31). However, there was an increase in the number of YLDs by 55.19% (95% UI: 49.57 to 60.73) (Table 2), due to population growth and aging of Brazil.

Among the FUs, in 2017, the rate of YLD of depressive disorders ranged from 442.9 per 100,000 (95% UI: 315.81–600.09) in Pará to 638.29 (95% UI: 447.04–868.23) in Santa Catarina (Table 2). There was no difference among the YLD rates of the FUs.

**Fig. 1:**
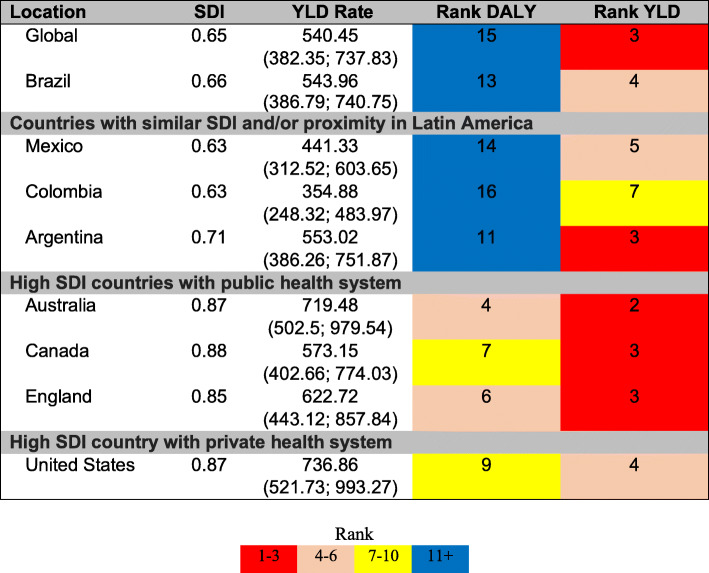
Socio-demographic Index, rate and ranking of DALY and YLD, worldwide, Brazil and other countries, GBD-2017

These disorders ranked 4th and 13th as leading causes of YLD and DALY, respectively, in Brazil, in 2017. The burden of depressive disorders was similar among the countries, YLD ranging from the 2nd position in Australia to 7th position in Colombia. However, as to DALY, there was greater variability and countries with similar SDI in Latin America ranked lower than the high SDI countries. Depressive disorders in the DALY ranged from Australia (4th) to Colombia (16th) (Fig. 1).

All countries presented age-standardized YLD rates similar to the overall rate, with UI overlaps. Among the countries analyzed, only Colombia had a statistically lower YLD rate than that of Australia and USA (Fig. 1).

**Fig. 2:**
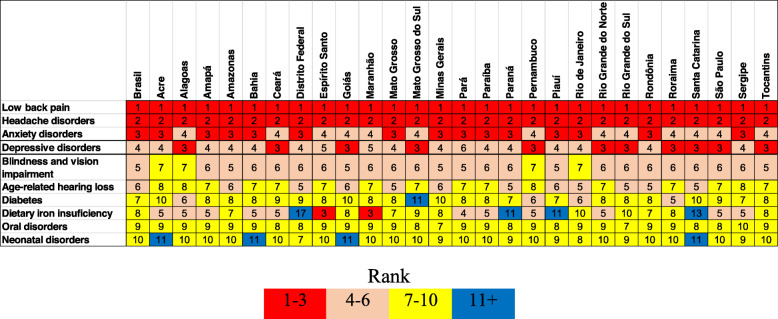
Leading ten causes of YLDs in Brazil and Federative Units, both sexes, GBD-2017

The classification of the ten leading causes of YLD in Brazil and FUs, in both sexes, is presented in Fig. 2, with depressive disorders ranking 3rd to 6th in Brazil and in all FUs.

**Fig. 3:**
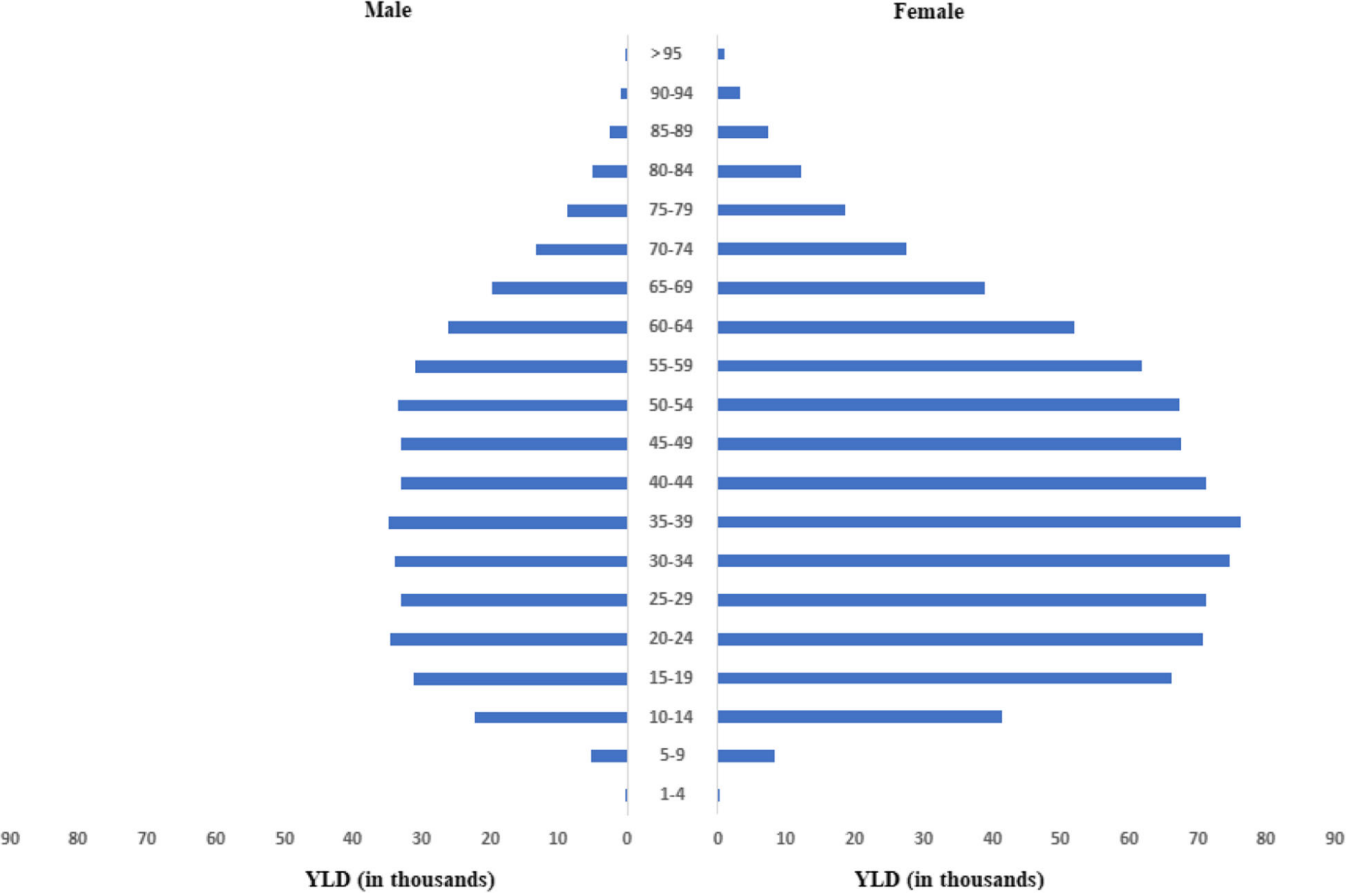
Number of YLD by age groups and sex for depressive disorders, Brazil, GBD-2017

Figure 3 shows the composition of the absolute numbers by age and sex for depressive disorders in GBD-2017. YLDs were considerably higher in women (837.269 95% UI: 593,162–1,140,266) compared to men (402,582 thousand 95% UI: 287,454–547,636). In both sexes, the highest proportion of YLDs was concentrated in the age groups in which people are active, between 15 and 64 years old (678,556 YLDs), followed by the age groups 65 years and over (108,870 YLDs) and 1 to 14 years, with 50,023 YLDs.

**Fig. 4:**
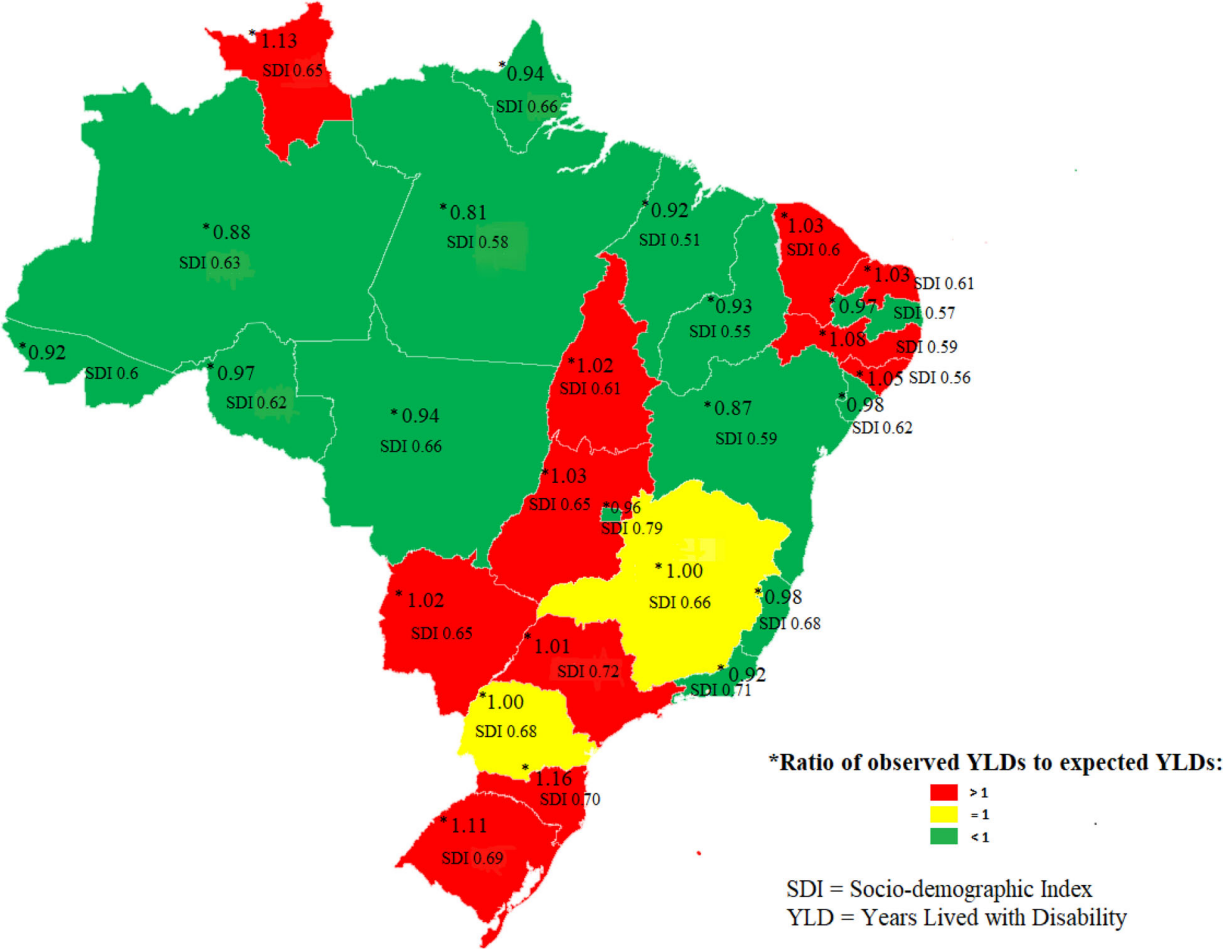
Ratio of observed/expected YLD for depressive disorders, given the socio-demographic index, Brazil and states, GBD-2017

Regarding the ratio of the observed and expected age-standardized YLD rates, based on SDI, for Minas Gerais and Paraná, YLD rates were equal to the expected rates. In 11 of the 27 FUs, YLD rates higher than expected were observed, varying from 1.01 in São Paulo to 1.16 in Santa Catarina; while in 14 FUs, YLD rates were lower than expected, with values between 0.81 in Pará and 0.98 in Espírito Santo and Sergipe (Fig. 4).

## Discussion

In study GBD-2017, depressive disorders were among the four major causes of disability in Brazil, following low back pain, headache, and anxiety disorders, and accounted for a high number of years lived with disability, especially in women, working-age adults, and residents of the Southern region of the country. Despite the decrease in the age-standardized rate of burden associated with depressive disorders in the last 27 years, there was a considerable increase in the number of YLD, which means an enlargement in demand for services and requires greater efforts in public health to provide adequate assistance to the Brazilian population.

There were significant differences in the prevalence of depression among some Brazilian FUs. Due to its large territorial extension, the investigation of prevalence of depression in all regions of the country is fundamental, since regional differences can be associated with variations in the rates of these disorders [31]. PNS-2013 found a significantly higher point prevalence of individuals at greater risk of depression in the Southern region (4.8%, 95% CI 4.1–5.4) and lower in the Northern region (2.9%, 95% CI 2.4–3.3) using the Patient Health Questionnaire-9 (PHQ-9) [8]. When considering self-report of prior medical diagnosis of depression, the prevalence was 12.6% (95% CI 11.2–13.9) in the South and 3.1% (95% CI 2.7–3.5) in the North of the country [32]. This result is corroborated by other studies that used the same measuring instrument and found a prevalence of depressive symptoms of 20.4% (95% CI 18.9–21.8) in Rio Grande do Sul [33] and 7% (95% CI 6–8) in Amazonas [34].

In the study GBD-2017, in addition to the high prevalence found in the Southern states, the states of Roraima (3.67), in the Northern region, and Pernambuco (3.52) and Alagoas (3.48), in the Northeastern, stood out. Comparability of these data is a complex task, since the estimates of GBD study are based not only on raw data of the studies available but also on adjustments based on covariables and other procedures [35]. Furthermore, there is a scarcity of studies for the Northern and Northeastern regions of the country [9].

It is worth mentioning that the states with the highest prevalence of depression also present with the highest mortality rates from suicide, according to data from studies showing depressive disorders are among the major risk factors for suicide deaths [4]. Rio Grande do Sul has the highest rate in the country: 10.5 per 100,000 (95% UI: 6.9–13). Roraima ranked 2nd, with a suicide rate of 9.4 per 100,000 (95% UI: 7.2–11.2) [36], indicating that there is still much to investigate on the subject. It also draws attention the fact that Pernambuco and Alagoas were the states with the highest mortality rates due to violence in 2015 [37, 38]; and violence is an important risk factor for onset and aggravation of depression [10].

Between 1990 and 2017, there was a considerable increase in the number of YLD for depression in Brazil. Therefore, greater attention must be given to mental health, since there are more people living with depression and the tendency is for it to increase as the population ages. Especially in developing countries such as Brazil, the increased life expectancy due to improved reproductive health, nutrition, and control of infectious diseases in childhood results in more people living until adulthood, the mean age of population increases, and the burden of disease shifts to noncommunicable and chronic diseases and disabilities, such as depression [30].

In Brazil, the epidemiological transition does not occur homogeneously among its regions; i.e., the less developed regions of the North and Northeast, with lower SDI, present a slower transition than the Southern and Southeastern regions [38]. Thus, the YLDs of the Southern states resemble those of high SDI countries, while the YLDs of most Northern states are closer to countries with medium SDI values. Although many FUs significantly improved as to fertility rates, income per capita, and mean years of education (SDI), there was an increase in the number of YLDs from depressive disorders in recent years, indicating a challenge for Brazilian mental health care.

It is known that the higher the SDI, the lower the mortality rates (YLL) for communicable, maternal, neonatal, and nutritional diseases [39]. Therefore, in high-income countries, such as Canada, Australia, and England, the most prevalent and disabling diseases, with lower mortality, such as depressive disorders, stand out and account for the highest positions in the DALY classification, whereas in low- and middle-income countries, such as Brazil, Mexico, Colombia, and Argentina, diseases with higher mortality rates still prevail [30].

Thus, the burden of disability (YLD) of depressive disorders does not seem to vary according to development of countries as measured by SDI, since MDD ranked among the ten major causes of YLD in 191 out of 195 countries analyzed by GBD-2016 [40]. In the GBD-2017 study, the YLD of these disorders in Brazil and in other countries, such as Mexico and Argentina, considered middle-income countries, was similar to the burden of high-income, politically and economically stable countries, like the USA, Canada, England, and Australia.

The lack of a relation between the YLD of depressive disorders and SDI may be associated to a limitation of GBD estimates, which refers to the scarcity of epidemiological data, especially in low- and middle-income countries and in places with subnational estimates, such as Brazil, hindering confidence in variations of prevalence and burden.

Moreover, the lack of a standard that allows predicting the burden of depressive disorders as a function of SDI may suggest that the burden of these disorders depends on factors other than those measured through SDI. It is, therefore, necessary to better understand the relation of SDI with the epidemiological factors of these disorders, at the individual level, before any interpretation.

It is a fact that social inequalities in income and education levels, included in SDI, are risk factors for depression, as revealed by a meta-analysis involving 56 studies from different countries [41]. In the National Health Survey (PNS-2013), depression was also associated with low levels of education [8], although its relation with income and fertility rates was not evaluated.

SDI allows monitoring not only the development of countries/regions over time but also calculating the expected estimates for each region, given its level of development [30]. In Brazil, the disability generated by depressive disorders was within expectation, given the SDI. In most of the Southern FUs, the observed YLD rate was higher than expected, while in most FUs in the North and Northeast, the opposite happened. Thus, although Southern states have better access to treatment [42], the impact of YLD on depressive disorders was greater in this region, as found in countries with high SDI [30]. In Brazil, 78.8% of individuals with depressive symptoms receive no type of treatment for this problem; in that, the Northern region has the largest proportion of untreated individuals (more than 90%), and the Southern region, the lowest proportion (67.5%) [42].

The results of this study corroborate findings from different regions of the world of depression affecting predominantly women [43–48], for reasons related to both biological and social factors [48, 49]. Regarding age, it is worrying that the burden of disability of these disorders is greater precisely in the working-age population, since depression is related to an important loss of productive potential, causing these people to be away from work [50]. In the USA, the prevalence of depression is increasing more rapidly among younger people, which may, over time, reduce the prevalence gradient differences between age groups [51]. These data show the urgency of investing mainly in preventive actions, early detection, and improving quality of services available for treatment of depression, focusing on the risk factors and predictors that may influence the prevalence and burden of these disorders [52].

Among the strengths of GBD study is the addition of covariates that best predict prevalence, the expansion of epidemiological data on mental disorders, and the improvement of subnational estimates. Regarding Brazil, as well as other countries, it is important to evaluate the need to include covariate on child sexual abuse and intimate partner violence in the depression estimates model of GBD, considering that interpersonal violence is one of the major causes of burden of disease in the country [37, 38].

There is also a need for ongoing studies with sustainable population data, which allow assessing the prevalence of depression in FUs, as well as identifying demographic subgroups that require more interventions. Trends of past-year depression from 2005 to 2015 in the US study [51] indicated the overall prevalence of depression increased significantly over this period, mainly due to stress, related to lack of employment, and low income. Brazil is currently experiencing one of the biggest economic crisis in its history, with 13.7 million unemployed [53], which is likely to affect the scenario of estimates of the burden of depressive disorders in the coming years.

In terms of GBD limitations to estimate the burden of mental disorders, including depressive disorders, it must be emphasized that the low coverage of epidemiological data on mental health, as previously mentioned, especially in less developed regions, such as Brazil [38, 44], makes the real contribution of these disorders to the global burden of disease still underestimated. In primary care and other general medical services, it is estimated that 30 to 50% of cases are undiagnosed [54], meaning the challenge of ensuring an increase in the population’s healthy life expectancy is greater than expected.

In addition, the distribution of severity levels of MDD and dysthymia was derived from a limited number of sources of data from high-income countries, which limited the overall representativeness of distribution of disorder severity. There is, therefore, a need for further studies with comparable methods in the distribution of MDD and dysthymia severity, and their variation among countries and levels of access to care [4].

## Conclusion

Increasingly more people are living with disability and limitations resulting from depressive disorders in Brazil, which demands greater funds for mental health to increase supply of prevention, early detection, and treatment programs, with sufficient quality and quantity. Currently, Brazil spends in mental health less than half of what is advocated by the World Mental Health (WHO) [55]. This challenge becomes even greater in the current political and economic context of the country, which has approved a Proposed Amendment to the Constitution (*Proposta de Emenda Constitucional*—PEC 55/2016), restricting funds allocated to the health sector over the next 20 years [56].

We must also be aware of the particularities of each state in order to direct resources to the areas with greatest need. To this end, the elucidation of epidemiological aspects of depressive disorders throughout the national territory, as presented in this study, is crucial. Furthermore, it provides data to expand the discussion about distribution and aspects related to these disorders in each locality, which still are scarce in the country.

## Data Availability

Data we used in this article are publicly available online on the official website of Institute of Health Metrics and Evaluation (http://ghdx.healthdata.org/gbd-results-tool).

## Abbreviations

BHAS: Bambuí Health Ageing Study
BHS: Bambuí Health Survey
CAMDEX: Cambridge Mental Disorders of the Elderly Examination
CAMGOG: Brief neuropsychological testing (cognitive section of the CAMDEX)
CDI: Children’s Depression Inventory
CES-D: Center for Epidemiologic Studies Depression Scale
95% CI: Confidence Interval 95%
D-10: Brief instrument for screening of depressive disorders in elderly people
DALY: Disability adjusted life years
DAWBA: Development and Well-Being Assessment
DSM-IV-TR: Diagnostic and Statistical Manual of Mental Disorders
FUs: Federated units
GBD: Global Burden of Disease Study
GDS-30: Geriatric Depression Scale
GHQ-12: General Health Questionnaire
GMS: Geriatric mental status
ICD-10: International Statistical Classification of Diseases and Health Related Problems
IHME: Institute for Health Metrics and Evaluation
MDD: Major depressive disorder
MDE: Major depressive episode
MINI: Mini-International Neuropsychiatric Interview
MG: Minas Gerais
MMSE: Mini-Mental State Examination
NPI: Neuropsychiatric Inventory
PEC: *Proposta de Emenda Constitucional* (Proposed Constitutional Amendment)
PHQ-9: Patient Health Questionnaire
PNAD: *Pesquisa Nacional por Amostra de Domicílios* (National Household Sample Survey)
PNS: *Pesquisa Nacional de Saúde* (National Health Survey)
PR: Paraná
SDI: Socio-demographic index
RGS: Rio Grande do Sul
RJ: Rio de Janeiro
SDQ: Strengths and Difficulties Questionnaire
SP: São Paulo
95% UI: 95% uncertainty intervals
WHS: World Health Survey
USA: United States of America
WHO: World Mental Health
YLD: Years lived with disability
YLL: Years of life lost
